# Antitumor Effects of a Sesquiterpene Derivative from Marine Sponge in Human Breast Cancer Cells

**DOI:** 10.3390/md19050244

**Published:** 2021-04-26

**Authors:** Li-Yuan Bai, Jui-Hsin Su, Chang-Fang Chiu, Wei-Yu Lin, Jing-Lan Hu, Chia-Hsien Feng, Chih-Wen Shu, Jing-Ru Weng

**Affiliations:** 1 Division of Hematology and Oncology, Department of Internal Medicine, China Medical University Hospital, Taichung 40447, Taiwan; lybai6@gmail.com (L.-Y.B.); d5686@mail.cmuh.org.tw (C.-F.C.); annavsbelle@yahoo.com.tw (J.-L.H.); 2 College of Medicine, China Medical University, Taichung 40402, Taiwan; 3 National Museum of Marine Biology and Aquarium, Pingtung 94450, Taiwan; x2219@nmmba.gov.tw; 4 Cancer Center, China Medical University Hospital, Taichung 40415, Taiwan; 5 Department of Pharmacy, Kinmen Hospital, Ministry of Health and Welfare, Kinmen 89142, Taiwan; u8557006@gmail.com; 6 Department of Fragrance and Cosmetic Science, College of Pharmacy, Kaohsiung Medical University, Kaohsiung 80708, Taiwan; chfeng@kmu.edu.tw; 7 Institute of Biopharmaceutical Sciences, National Sun Yat-sen University, Kaohsiung 80424, Taiwan; cwshu@g-mail.nsysu.edu.tw; 8 Department of Marine Biotechnology and Resources, National Sun Yat-sen University, Kaohsiung 80424, Taiwan; 9 Doctoral Degree Program in Marine Biotechnology, National Sun Yat-sen University, Kaohsiung 80424, Taiwan; 10 Graduate Institute of Natural Products, College of Pharmacy, Kaohsiung Medical University, Kaohsiung 80708, Taiwan

**Keywords:** ilimaquinone, sponge, cell cycle arrest, apoptosis, autophagy, breast cancer

## Abstract

In this study, the anti-proliferative effect of ilimaquinone, a sesquiterpene derivative from the marine sponge, in breast cancer cells was investigated. Ilimaquinone inhibited the proliferation of MCF-7 and MDA-MB-231 breast cancer cells with IC_50_ values of 10.6 μM and 13.5 μM, respectively. Non-tumorigenic human breast epithelial cells were less sensitive to ilimaquinone than breast cancer cells. Flow cytometric and Western blot analysis showed that ilimaquinone induced S-phase arrest by modulating the expression of p-CDC-2 and p21. Ilimaquinone induces apoptosis, which is accompanied by multiple biological biomarkers, including the downregulation of Akt, ERK, and Bax, upregulation of p38, loss of mitochondrial membrane potential, increased reactive oxygen species generation, and induced autophagy. Collectively, these findings suggest that ilimaquinone causes cell cycle arrest as well as induces apoptosis and autophagy in breast cancer cells.

## 1. Introduction

Breast cancer has the highest incidence rate (73.2 per 100,000) among cancers in women. In 2012, there were 1.7 million new cases of breast cancer globally, which accounted for 25% of all cancers [1,2]. Currently, there are 324,000 deaths due to breast cancer, which is the most common cause of death in under developed countries in 2012 [3]. Major risk factors for breast cancer include (but are not restricted to) hormone imbalance, obesity, alcohol consumption, and genetic mutations [3]. In addition to surgery, chemotherapy, radiotherapy, hormone therapy, targeted therapy, and immunotherapy are the available treatments for breast cancer. However, the treatment of breast cancer ranges from 30,000 to 60,000 estimates by stage, which is an economic burden for patients [4]. Despite the cost, the overall survival rate of breast cancer with chemotherapy is still below 34 months [5]. This highlights the urgent need for the development of new therapeutic strategies.

Natural products have served as an enriched source to provide potential drugs for centuries. Some natural products represent various pharmacological effects that are used to treat several diseases. For example, paclitaxel from *Taxus brevifolia* has been approved by the FDA for treating breast cancer and ovarian cancer since 1992 [6]. Recently, secondary metabolites from marine organisms have garnered considerable attention owing to their therapeutic application in cancer therapies [7]. Previous studies have shown that polysaccharides from red seaweed inhibit cell growth by inducing apoptosis in various cancer cell lines, including breast cancer [8,9]. Cytarabine (Ara-C), an analog of natural arabino nucleosides and cytosine arabinose, has been used clinically to treat leukemia by interrupting DNA synthesis for several years [10]. In a Phase III clinical trial, it is worth noting that improved disease control was achieved with trabectedin (a synthetic derivative from tunicate) in both liposarcoma and leiomyosarcoma patients [11]. Furthermore, Zalypsis^®^, a synthetic alkaloid structurally related to renieramycin J from marine sponges, has been reported to have anti-leukemia properties and synergistic effects with chemotherapeutic agents, including cytarabine and daunorubicin [12].

**Figure 1:**
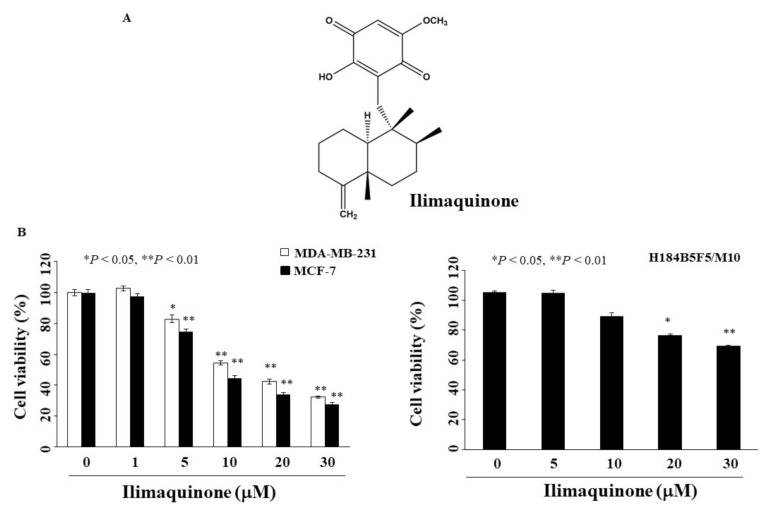
Antiproliferative effects of ilimaquinone on breast cancer and normal human breast epithelial cells (**A**). Chemical structure of ilimaquinone, (**B**) Breast cancer cells (MCF-7 and MDA-MB-231) and normal human breast epithelial cells (H184B5F5/M10) were treated with ilimaquinone at the indicated concentrations for 48 h, and cell viability was detected by MTT assays. *Points* represent means; *bars* represent S.D. (*n* = 3–6). * *p* < 0.05, ** *p* < 0.01 compared with the control group.

Ilimaquinone, a sesquiterpene derivative, was originally found in marine sponges (*Halichondria* sp.) [13]. It has been reported that ilimaquinone induces Golgi fragmentation with anti-viral activity [14]. In addition to being a Golgi disruptor, the anti-tumor activity of ilimaquinone has drawn attention from many investigators. Multiple studies have reported that ilimaquinone induces apoptosis in various human cancer cell lines, including prostate, colon, breast, and multiple myeloma [13,15,16,17]. For example, p53 activation and ROS–ERK/p38 MAPK–CHOP signaling contributed to ilimaquinone-induced apoptosis in human colon cancer cells [16]. Park et al. reported that ilimaquinone suppressed cell growth by downregulating β-catenin in multiple myeloma cells [17]. We found that ilimaquinone induced apoptosis and autophagy through p53 activation in oral cancer cells [18]. However, its anti-tumor activity in breast cancer remains unclear. Therefore, in this study, we examined the anti-tumor effect of ilimaquinone (Figure 1A) in breast cancer cells and found that this compound causes S phase arrest, apoptosis, ROS generation, and autophagy.

## 2. Results

### 2.1. Ilimaquinone Inhibits Viability of Breast Cancer Cells

To determine the anti-proliferative activity of ilimaquinone, two breast cancer cell lines, MCF-7 and MDA-MB-231, were examined using MTT assays. As shown in Figure 1B, the concentrations of ilimaquinone to inhibit cell growth by 50% after 48 h were 10.6 μM and 13.5 μM in MCF-7 and MDA-MB-231 cells, respectively. The viability of ilimaquinone in non-tumorigenic human breast epithelial cells (H184B5F5/M10) was also assessed. After treatment with ilimaquinone for 48 h, this compound was less sensitive to H184B5F5/M10 cells with an IC_50_ value higher than 30 μM (Figure 1B). As the IC_50_ value of MCF-7 cells was lower than that of MDA-MB-231 after treatment with ilimaquinone, the former cell line was used in subsequent experiments.

### 2.2. Ilimaquinone Induces S Phase Arrest in MCF-7 Cells

**Figure 2:**
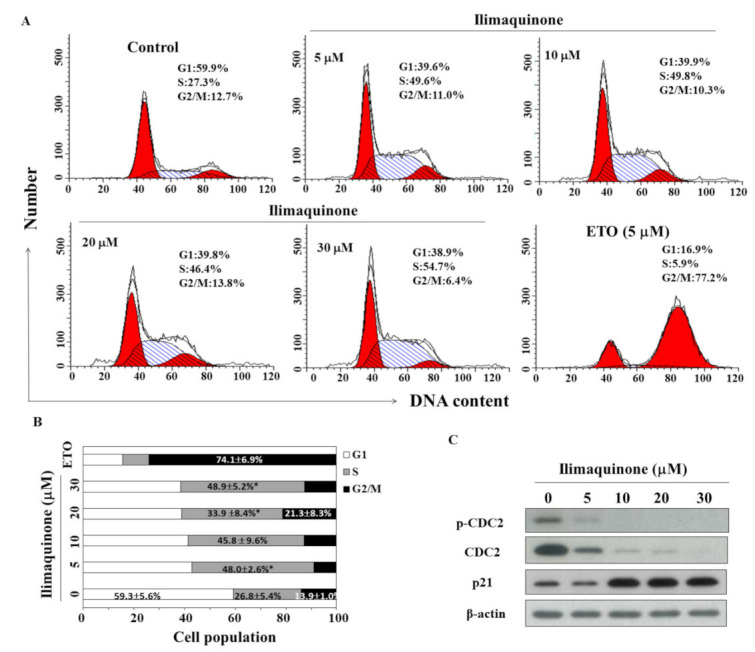
Effects of ilimaquinone on cell cycle and cell cycle-related proteins in MCF-7 cells. (**A**) Flow cytometric analysis of the effect of ilimaquinone for 48 h in MCF-7 cells. (**B**) The percentage of cells in each cell cycle phase was determined by PI staining and analyzed by flow cytometry. Values are means ± S.D. of three independent experiments. * *p* < 0.05 compared with the control group. (**C**) Western blot analysis of the phosphorylation/expression of CDC2 and p21 after the treatment of ilimaquinone for 48 h in MCF-7 cells.

To study the effect of ilimaquinone on the progression of cell cycle, MCF-7 cells were treated with ilimaquinone for 48 h and stained with propidium iodide (PI). Flow cytometric analysis demonstrated that ilimaquinone increased the cell population in the S phase (Figure 2A, etoposide as a positive control). As shown in Figure 2B, the cell population in the S phase was increased from 26.8 ± 5.4% in the control group to 48.9 ± 5.2% in the 30 μM ilimaquinone group. Western blot analysis demonstrated that ilimaquinone downregulated the level of cell division cycle (CDC)2 at both phosphorylation and total form, which was accompanied by the upregulation of p21 expression (Figure 2C).

### 2.3. Ilimaquinone Induces Apoptosis in MCF-7 Cells

**Figure 3:**
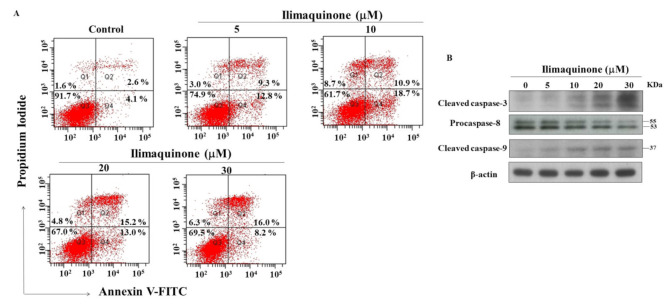
Ilimaquinone induces apoptosis in MCF-7 cells. (**A**) Effect of ilimaquinone on propidium iodide (PI)/annexin V staining at 48 h. (**B**) Concentration-dependent effect of ilimaquinone on cleaved caspase-3, procaspase-8, and cleaved caspase-9 in MCF-7 cells after 48 h exposure.

Apoptosis is one of the mechanisms of cell death following exposure to radiation and chemotherapy [19], and we further examined the role of apoptosis in ilimaquinone-induced cell death. PI/Annexin V analysis showed that number of apoptotic cells increased after treatment with ilimaquinone for 48 h in MCF-7 cells (Figure 3A). The double-stained (Annexin V+/PI+) cells were increased from 2.6% (the control group) to 16.0% after the treatment of ilimaquinone at 30 μM in a dose-dependent manner (Figure 3A). The percentage of (Annexin V-/PI+) cells were increased from 4.1% in the control group to 18.7% in the 10 μM ilimaquinone group (Figure 3A). It is notable that the necrotic cells were marginally increased after treatment with ilimaquinone, which suggested that necrosis might be involved in ilimaquinone-induced cell death. Western blotting demonstrated that ilimaquinone upregulated both the levels of the cleaved caspase-3 and caspase-9 [20] while downregulating the expression of procaspase-8 [21] (Figure 3B). The above results revealed that ilimaquinone induces cell death in MCF-7 cells, mainly through apoptosis.

### 2.4. Ilimaquinone Modulates Pro-Apoptotic Biomarkers and Mitochondrial Membrane Potential (Δψm) in MCF-7 Cells

**Figure 4:**
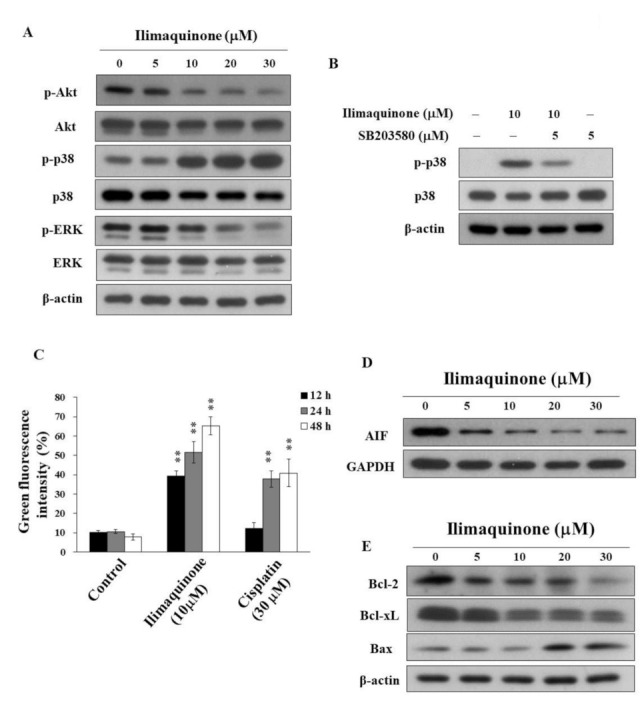
Effects of ilimaquinone on the levels of various apoptosis signaling effects in MCF-7 cells (**A**). Concentration-dependent effects of ilimaquinone on the phosphorylation of Akt, p38, and ERK in MCF-7 cells. (**B**) Phosphorylation/expression of p38 in ilimaquinone (10 μM) alone or in combination of the p38 inhibitor SB203580 (5 μM) for 48 h in MCF-7 cells. (**C**) Effect of mitochondrial membrane potential (Δψm) after the treatment of 10 μM ilimaquinone or 30 μM cisplatin for 48 h in MCF-7 cells. Δψm was assessed with green–red fluorescent probe JC-1. (*n* = 3). Data are represented as means ± S.D. ** *p* < 0.01. (**D**) Effect of ilimaquinone on AIF expression in MCF-7 cells. (**E**) Expression of Bcl-2, Bcl-xL, and Bax in ilimaquinone -treated cells for 48 h.

The activation of Akt contributes to the molecular pathogenesis of breast cancer, and it is associated with tumorigenesis and drug resistance [22]. Avivar-Valderas et al. reported that the some signaling factors, such as p38, JNK, and ER stress-responsive molecules, are involved in mammary gland development and breast tumor growth [23]. To better understand the mechanisms of ilimaquinone on cell proliferation and apoptosis, we investigated the role of Akt and MAPK signaling pathways. As shown in Figure 4A, ilimaquinone suppressed the phosphorylation of Akt and ERK in a concentration-dependent manner, while it was accompanied by a parallel increase in p-p38 in MCF-7 cells. In order to confirm that ilimaquinone upregulates p38, a p38 inhibitor, SB203580, was added and assessed using Western blotting and MTT assays. As shown in Figure 4B, compared with ilimaquinone-treated MCF-7 cells, the phosphorylation level of p38 was less extent after the combination of SB203580. However, exposure to SB203580 did not alter ilimaquinone-induced cytotoxicity, suggesting that the activation of p38 may not be the major target related to ilimaquinone-induced cell death in MCF-7 cells (data not shown).

Furthermore, MCF-7 cells were treated with DMSO or ilimaquinone for 48 h, stained with the green-red fluorescent probe JC-1, and analyzed for mitochondrial membrane potential (Δψm). JC-1 aggregates (red fluorescence) with high Δψm in intact cells. In response to the loss of Δψm, green fluorescence was observed. Consistent with the ability to induce apoptosis, ilimaquinone increased the green fluorescence intensity in a time-dependent manner in MCF-7 cells, suggesting the loss of Δψm and mitochondrial damage (Figure 4C, cisplatin as a positive control). In addition, ilimaquinone decreased the level of apoptosis-inducing factor (AIF), which is a highly conserved protein that is confined to the mitochondrial intermembrane [24] in the cytosol of MCF-7 cells (Figure 4D). Furthermore, Western blotting showed that ilimaquinone decreased the expression of Bcl-2 and Bcl-xL, which is accompanied by parallel increases in the proapoptotic protein Bax (Figure 4E).

### 2.5. Ilimaquinone Increases Reactive Oxygen Species (ROS) Generation in MCF-7 Cells

**Figure 5:**
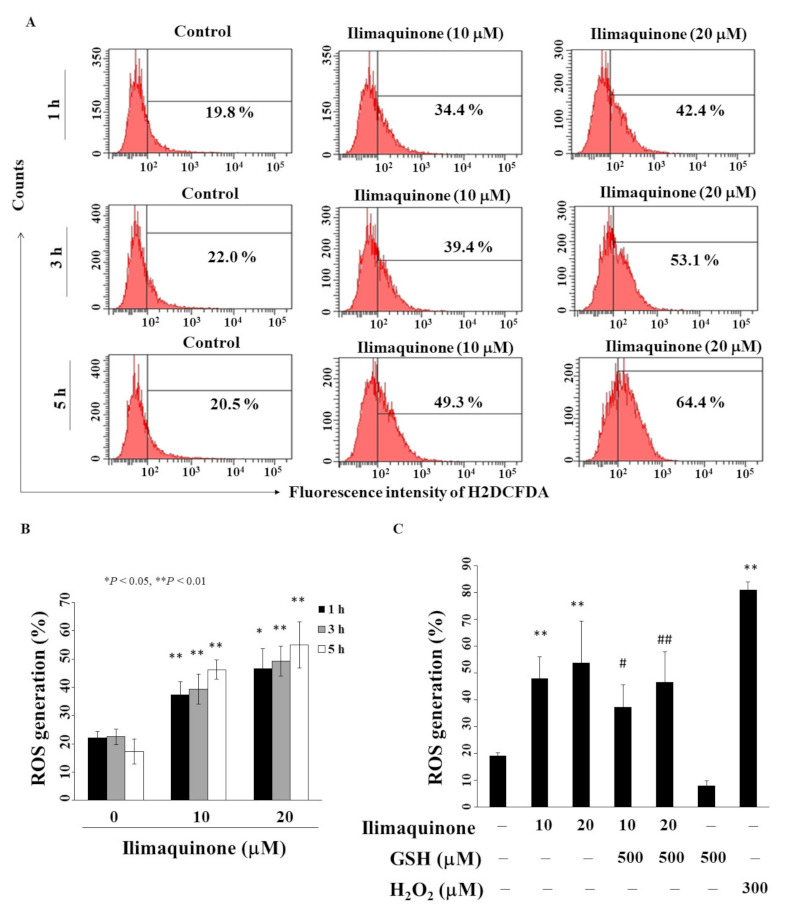
Ilimaquinone increases ROS generation in MCF-7 cells. (**A**) Cells were treated with DMSO or ilimaquinone at 1, 3, and 5 h. (**B**) Histogram of ROS production of ilimaquinone at the indicated time interval in MCF-7 cells. *Columns* represent means; *bars* represent S.D. * *p* < 0.05, ** *p* < 0.01 compared with the control group. (**C**) Statistical analysis of ROS generation after the treatment of ilimaquinone alone or in the combination of 500 μM glutathione (GSH) or 300 μM H_2_O_2_ alone for 3 h (*n* = 5). Data are represented as means ± S.D. ** *p* < 0.01 compared with the control group. # denotes *p* < 0.05 when compared with ilimaquinone (10 μM) alone group. ## denotes *p* < 0.05 when compared with ilimaquinone (20 μM) alone group.

Previous studies revealed that an imbalance in ROS production mediates cancer initiation and promotion in multistep carcinogenesis, including breast cancer [25,26]. We found that ilimaquinone increased ROS generation in a concentration- and time-dependent manner in MCF-7 cells (Figure 5A). As shown in Figure 5B, compared with the control group, the percentage of ROS generation was increased from 22.5% to 49.3% after treatment with 20 μM ilimaquinone for 3 h. Pretreatment with antioxidant glutathione (GSH) for 15 min could partially rescue ilimaquinone-induced ROS generation (Figure 5C, H_2_O_2_ as positive control).

### 2.6. Ilimaquinone Induces Autophagy in MCF-7 Cells

**Figure 6:**
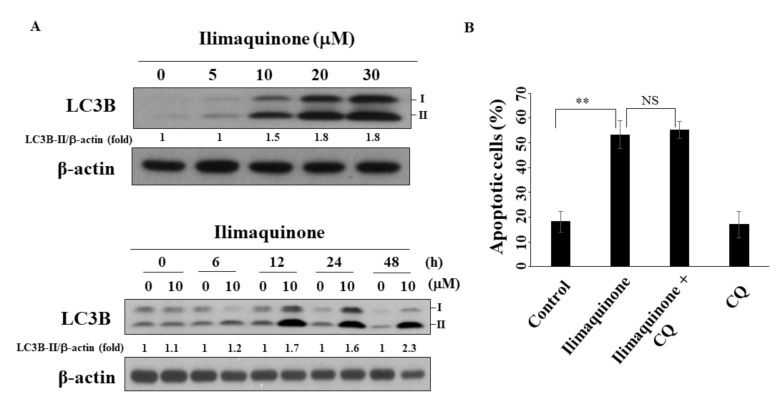
Ilimaquinone induces autophagy in MCF-7 cells. (**A**) Upper panel, the formation of LC3B in MCF-7 cells treated with ilimaquinone for 48 h. Lower panel, the formation of LC3B after the treatment of ilimaquinone (10 μM) in MCF-7 cells. LC3B-II was quantified as LC3B-II/β-actin ratio. (**B**) The percentage of apoptotic cells of ilimaquinone (10 μM) or in combination with chloroquine (CQ, 10 μM) in MCF-7 cells. *Points* represent means; *bars* represent S.D. (*n* = 3). ** *p* < 0.01, NS denotes no significance.

Several reports have demonstrated the decisive role of autophagy in the progression and treatment of breast cancer [27,28]. As shown in Figure 6A, ilimaquinone increased the formation of LC3B-II in a concentration- and time-dependent manner in MCF-7 cells. To further examine the role of autophagy in ilimaquinone-induced apoptosis, the autophagic inhibitor chloroquine (CQ) was used. The results showed that the combination of CQ had no obvious effect on the percentage of ilimaquinone-induced apoptotic cells. This suggested that autophagy may be separate from ilimaquinone-mediated cell death.

## 3. Discussion

Accumulating evidence indicates that natural products are useful sources of secondary metabolites, and they play an important role in cancer therapy [7,29]. It has been reported that ilimaquinone can inhibit the growth of various cancer cells [13,15,16,17,18]. In this study, ilimaquinone induced cell cycle arrest, apoptosis, ROS generation, and autophagy in MCF-7 cells. Compared with the breast cancer cells, ilimaquinone had a minimal effect on non-tumorigenic human breast epithelial cells H184BF5/M10 below 10 μM (Figure 1B).

Several studies have revealed that marine natural products inhibit cell growth in prostate cancer and lung cancer by targeting microtubules [30,31]. Hood et al. reported that peloruside A, a macrolide isolated from marine sponge, causes G2/M arrest by promoting microtubule polymerization in lung cancer cells [30]. Eribulin, a synthetic product of halichodrin B isolated from marine sponge, inhibits microtubule polymerization and led to G2/M arrest in breast cancer cells [32]. Moreover, Rokkaku et al. showed that fucoxanthinol inhibited osteosarcoma cells by inducing G1 arrest by downregulating cyclin-dependent kinase (CDK)4, CDK6, and cyclin E [33]. The results of the present study demonstrated that ilimaquinone induced S phase arrest, which was accompanied by the downregulation of cell cycle-regulated proteins, including p-CDC2 and p-CDC25C. The activation of CDC2 is an essential step for mitosis entry, and the accumulation of the inactive Tyr15 phosphorylated cyclin B/CDC2 occurred at the G2 checkpoint [34,35]. It is well known that CDK inhibitor p21 binds CDK, regulating the G1 to S phase transition of the cell cycle [36]. The expression of p21 could be regulated by the transcription factor p53 or DNA damage agents, and the overexpression of p21 inhibits the proliferation of mammalian cells [37,38]. In the present study, ilimaquinone increased the p21 protein level in a dose-dependent manner, suggesting that p21 might contribute to ilimaquinone-induced S arrest.

In addition to cell cycle arrest, apoptosis is one of the major factors for regulating cell proliferation. Do Nascimento-Neto et al. reported that halilectin-3 induced apoptosis through caspase-9 activation in breast cancer cells [39]. Our results showed that ilimaquinone increased the cleavage of caspase-3 and caspase-9 and decreased the expression of procaspase-8. We also found that this compound induced mitochondrial membrane potential collapse and decreased the expression of AIF. These results suggested that ilimaquinone induced apoptosis through mitochondrial and extrinsic pathways, which is consistent with other studies [13,18]. Karanam et al. reported that a cyclic dipeptide from marine sponges inhibited cell growth through mitochondrial dysfunction and downregulating Akt signaling in hepatocellular carcinoma cells [40]. It has been reported that activated Akt and ERK signaling pathways are involved in breast cancer progression [41]. In the present study, we observed that the phosphorylation of Akt was downregulated after treatment with ilimaquinone. The overexpression of p38 was found in highly metastatic human tumors and was associated with metastatic phenotypes of breast tumor samples [42]. Do et al. found that ilimaquinone inhibited cell growth by increasing p-p38 and p-ERK in colon cancer cells [13]. However, our results demonstrated that ilimaquinone inhibited p-ERK and increased p-p38 in MCF-7 cells. We assume that this discrepancy could be attributed to the different cell lines used. We also examined the morphology of cell–cell adhesion in ilimaquinone-treated cells by immunofluorescence. The results showed that the level of membrane-bound E-cadherin was increased after the treatment of ilimaquinone, while the level of nuclear β-catenin was decreased (Figure S1), supporting the notion that ilimaquinone inhibited cell proliferation in breast cancer cells. Nevertheless, the detailed mechanisms of ilimaquinone on the E-cadherin/β-catenin complex will need further investigation.

Previous studies showed that ROS generation in cancer contributed to inhibit cell proliferation, induce DNA damage, autophagy, and cell death [43,44,45]. Therefore, ROS generation is related to the anti-tumor effects of some chemotherapeutic agents [46,47]. For example, paclitaxel induced apoptosis through ROS production in breast cancer cells [46]. Kleih et al. reported that cisplatin caused mitochondrial ROS generation and apoptosis in ovarian cancer cells [47]. Our results showed that ilimaquinone-induced ROS generation and GSH effectively attenuated this phenomenon in MCF-7 cells.

Recently, autophagy has been recognized as one way to regulate cell death, and the impairment of autophagy is related to resistance to anticancer therapy [48]. For example, Bousquest et al. reported that autophagy inhibition can reverse resistance to drugs including cisplatin, paclitaxel, and epirubicin in breast cancer cells [49]. For Ara-C-resistant leukemia cells, autophagy enhanced Ara-C-induced cell death [50]. Previous studies have shown that ilimaquinone induces autophagy in colon cancer, glioblastoma cells, and oral cancer cells through p53 activation [16,18,51]. Similarly, we found that ilimaquinone increases the formation of the autophagic biomarker, LC3B-II, in a dose- and time-dependent manner in breast cancer cells. Interestingly, unlike the autophagy inhibitor-protected ilimaquinone-induced apoptosis in oral cancer cells [18], our results revealed that no obvious change in ilimaquinone-treated breast cancer cells.

In summary, we found that ilimaquinone was less sensitive to non-tumorigenic human breast epithelial cells than breast cancer cell lines. Ilimaquinone exhibits S-phase arrest, modulates apoptosis and autophagy, and increases ROS generation. These findings suggest that ilimaquinone has a potential therapeutic role in breast cancer treatment.

## 4. Materials and Methods

### 4.1. Reagents, Chemicals, Antibodies

Ilimaquinone was provided from one of the co-authors, Professor. Jui-Hsin Su (National Museum of Marine Biology and Aquarium), and the purity of this compound was provided as previously reported [52]. Antibodies to phospho-Akt (Ser^473^), Akt, phospho- p38 (^180/182^Thr/Tyr), p38, phospho-ERK (^202/204^Thr/Tyr), ERK, AIF, Bcl-2, Bcl-xL, Bax, cleaved caspase-3, cleaved caspase-9, LC3B, p62, p21, CDC-2, phospho-CDC2 (^15^Tyr), phospho-CDC25C (Ser^216^), and CDC25C were bought from Cell Signaling Technology (Danvers, MA, USA). Antibody to procaspase-8 was purchased from Millipore (Darmstadt, Germany). β–actin antibody was obtained from Sigma-Aldrich (Saint Louis, MO, USA). All chemicals were dissolved in dimethyl sulfoxide (DMSO and diluted to cells at a final DMSO concentration (0.1%).

### 4.2. Cell Culture

Both human breast cancer cell lines MCF-7 and MDA-MB-231 were purchased from the American Type Culture Collection (Manassas, VA, USA) and maintained in Dulbecco’s modified Eagles medium/Nutrient Mixture-F12 (DMEM/F12) (Invitrogen, Carlsbad, CA, USA). Non-tumorgenic human breast epithelial cell line (H184B5F5/M10) was kindly provided by one of the authors, Prof. Chih-Wen Shu (National Sun Yat-sen University) and maintained in DMEM (Invitrogen). All cells were maintained in a humidified incubator containing 5% CO_2_ at 37 °C.

### 4.3. Cell Viability Assay

Cells (5 × 10^3^) were incubated in 96-well plates for 24 h following added with drug or DMSO. Then, cell viability was determined by 3-(4,5-dimethylthiazol-2-yl)-2,5-diphenyltetrazolium bromide (MTT) solution (0.5 mg/mL) as described previously [53]. After removing the medium, DMSO was added. A Multiskan Go plate reader (ThermoFisher Scientific, Waltham, MA, USA) was used to calculate the absorbance.

### 4.4. Cell Cycle and Apoptosis Analysis

For apoptosis analysis, cells (2 × 10^5^) were treated with drug or DMSO for 48 h and then incubated with Annexin V–FITC and propidium iodide (PI) for 15 min according to the vender’s protocol (BD Pharmingen, San Diego, CA, USA). Then, cells were analyzed using BD FACSCanto (BD, Franklin Lakes, NJ, USA). For cell cycle analysis, the cells were stained with PI and analyzed using FACSCanto flow cytometric analysis.

### 4.5. Mitochondrial Membrane Potential (Δψm) and Reactive Oxygen Species (ROS) Generation

Mitochondrial membrane potential was determined using JC-1 dye according to the manufacture’s instruction. Briefly, cells were stained with JC-1 (2.5 μM) or 2′,7′-dichlorodihydrofluorescein diacetate (DCFH-DA) (5 μM) for Δψm or ROS determination, respectively. Then, cells were analyzed by fluorescence intensity using flow cytometry (ThermoFisher Scientific).

### 4.6. Western Blot Analysis

Lysates of DMSO- or drug-treated MCF-7 cells were prepared for immunoblotting of phospho-Akt (Ser^473^), Akt, phospho-p38 (Thr/Tyr^180/182^), p38, phospho-ERK (Thr/Tyr^202/204^), ERK, AIF, Bcl-2, Bcl-xL, Bax, cleaved caspase-3, cleaved caspase-9, procaspase-8, LC3B, p21, CDC-2, phospho-CDC2 (Tyr^15^), phospho-CDC25C (Ser^216^), CDC25C, and β–actin. Western blot analysis was performed as previously reported [53].

### 4.7. Statistical Analysis

All experiments were performed in triplicates. Statistical analyses of data were performed using Student’s *t*-test. * *p* < 0.05 was considered to be statistically significant.

## Data Availability

Data are contained within the article.

## Supplementary Materials

The following are available online at https://www.mdpi.com/article/10.3390/md19050244/s1, Figure S1: Localization analysis of E-cadherin and β-catenin in MCF-7 cells after ilimaquinone treatment.
