# Supplementary material for: Antitumor Effects of a Sesquiterpene Derivative from Marine Sponge in Human Breast Cancer Cells
Source: Mar Drugs. 2021 Apr 26;19(5):244. doi: 10.3390/md19050244 (PMC8144972; doi:10.3390/md19050244)
Supplement: Supplementary file 1 [file marinedrugs-19-00244-s001.zip › marinedrugs-1139168-SI.pdf]

Supporting Information

Antitumor Effects of a Sesquiterpene Derivative from Marine Sponge in Human Breast Cancer Cells

Li-Yuan Bai [1,2,†], Jui-Hsin Su [3,†], Chang-Fang Chiu [1,4], Wei-Yu Lin [5], Jing-Lan Hu [1], Chia-Hsien Feng [6], Chih-Wen Shu [7], and Jing-Ru Weng [8,9,10,*]

[1]Division of Hematology and Oncology, Department of Internal Medicine, China Medical University Hospital, Taichung 40447, Taiwan

[2]College of Medicine, China Medical University, Taichung 40402, Taiwan

[3]National Museum of Marine Biology and Aquarium, Pingtung 94450, Taiwan

[4]Cancer Center, China Medical University Hospital, Taichung 40415, Taiwan

[5]Department of Pharmacy, Kinmen Hospital, the Ministry of Health and Welfare, Kinmen 89142, Taiwan

[6]Department of Fragrance and Cosmetic Science, College of Pharmacy, Kaohsiung Medical University, Kaohsiung 80708, Taiwan

[7]Institute of BioPharmaceutical Sciences, National Sun Yat-sen University, Kaohsiung 80424, Taiwan

[8]Department of Marine Biotechnology and Resources, National Sun Yat-sen University, Kaohsiung 80424, Taiwan

[9]Doctoral Degree Program in Marine Biotechnology, National Sun Yat-sen University, Kaohsiung 80424, Taiwan

[10]Graduate Institute of Natural Products, College of Pharmacy, Kaohsiung Medical University, Kaohsiung 80708, Taiwan

Figure		Page
S1	Localization analysis of (A) E-cadherin and (B) β-catenin in MCF-7 cells after ilimaquinone (5 μM) treatment. Cells were incubated with DMSO or ilimaquinone (5 μM) for 48 h, then washed three with PBS, fixed with 4% paraformaldehyde (PFA) and immunostained by antibodies against E-cadherin and β-catenin. Bar: 100 μm.	4

Fig. S1

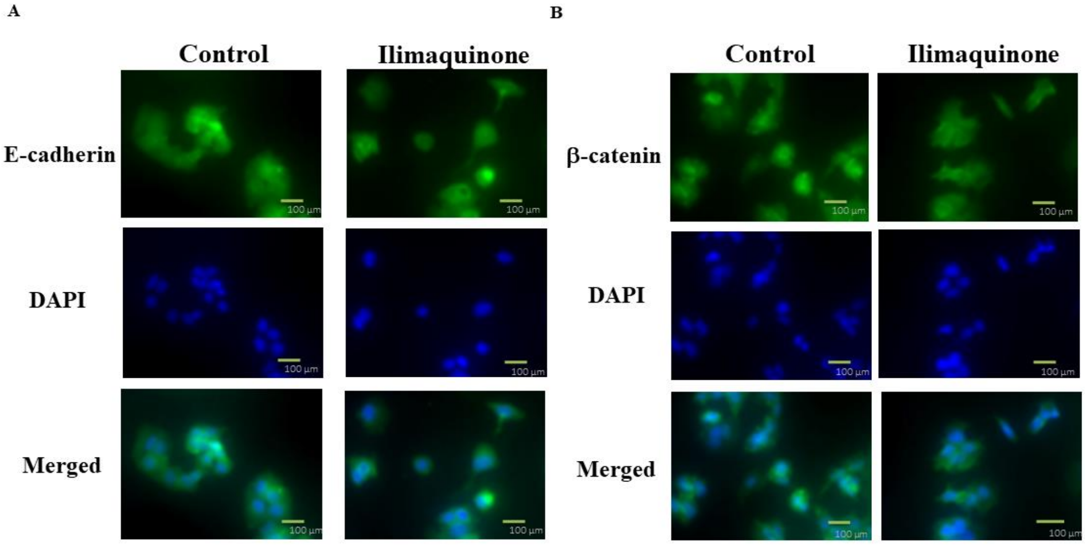
A
B
Control
Ilimaquinone
Control
Ilimaquinone
E-cadherin
β-catenin
DAPI
DAPI
Merged
Merged
100 µm
